# Effects of Dietary Ursolic Acid on Growth Performance and Intestinal Health of Largemouth Bass (*Micropterus salmoides*)

**DOI:** 10.3390/ani14172492

**Published:** 2024-08-27

**Authors:** Min Wang, Yongfang Wang, Xiang Li, Yue Yin, Xiwen Zhang, Shuang Wu, Hongquan Wang, Yurong Zhao

**Affiliations:** 1 College of Animal Science and Technology, Hunan Agricultural University, Changsha 410128, China; 13627411846@163.com (M.W.); 15107041055@163.com (Y.W.); lxffy0123@163.com (X.L.); 15665173374@163.com (Y.Y.); zxw577258@163.com (X.Z.); 2 Fisheries College, Hunan Agricultural University, Changsha 410128, China; 16670200044@163.com

**Keywords:** ursolic acid, growth performance, intestinal barrier, intestinal microbiota, largemouth bass

## Abstract

**Simple Summary:**

High-density intensive farming easily induces stress in largemouth bass, leading to significant impairment of growth performance and intestinal health. Ursolic acid (UA), a naturally abundant pentacyclic triterpenoid, has multifaceted biological activities, encompassing anti-inflammatory, antioxidant, lipid-modulating, and hypoglycemic properties. This study examined the effects of UA on growth performance and intestinal barrier in largemouth bass. Our findings demonstrate that dietary supplementation of UA significantly enhances the growth performance and intestinal antioxidant capacity in largemouth bass, while improving intestinal barrier function through its influence on the abundance of intestinal flora such as Tenericutes, Firmicutes, and Mycoplasma. Optimal dietary UA levels for largemouth bass were determined to be between 498 and 520 mg/kg. These findings serve as a valuable reference for largemouth bass production.

**Abstract:**

This study aimed to investigate the effects of ursolic acid (UA) on the growth performance and intestinal health of largemouth bass (*Micropterus salmoides*). Four diets were formulated with UA supplementation at 0, 250, 500, and 1000 mg/kg, defined as the control (CON), UA250, UA500, and UA1000, respectively. After an 8-week feeding experiment, the results showed that, in the UA500 group, the final body weight (FBW), weight gain rate (WGR), and specific growth rate (SGR) increased, and the feed conversion ratio (FCR) and hepatosomatic index decreased. Total superoxide dismutase (T-SOD) activity exhibited a significant increase, and malondialdehyde (MDA) content decreased. An intestinal histological analysis revealed an improvement in the intestinal structural integrity of the UA500 group. The mRNA relative expression levels of physical barrier-related genes [*occludin*, *claudin-1*, and zonula occluden-1 (*zo-1*)] were upregulated. The mRNA relative expression of interlenkin 10 (*il-10*) increased, and the mRNA relative expression of interlenkin 1β (*il-1β*) and tumor necrosis factor-α (*tnf-α*) significantly decreased. The abundance of Firmicutes and Proteobacteria decreased, and the abundance of Tenericutes increased. The abundance of *Mycoplasma*, *Cyanobium*, and *Staphylococcus* decreased, while the abundance of *Clostridium* increased. In conclusion, dietary supplementation of UA significantly enhanced the growth performance and antioxidant capacity of largemouth bass while improving intestinal barrier function through its influence on the abundance of intestinal flora, such as Tenericutes, Firmicutes, and *Mycoplasma*. Optimal dietary UA levels for largemouth bass were determined to be between 498 and 520 mg/kg based on quadratic regression analyses of WGR, SGR, and FCR or T-SOD and MDA content.

## 1. Introduction

High-density intensive aquaculture offers space-saving and profitability advantages, and it has become the prevailing trend in modern aquaculture. However, susceptibility to viruses, bacteria, and parasites, as well as compromised fish health, particularly intestinal health, is associated with high-density culture and high-energy feed intake [1,2]. The intestine plays a pivotal role in fish, as it serves dual functions: it is the primary site for nutrient digestion and absorption, and it is an indispensable component of the immune system. Consequently, preserving the health of the intestinal tract is imperative for ensuring the overall wellbeing and optimal growth of fish.

Ursolic acid (UA), a pentacyclic triterpenoid compound, is widely distributed in various plants such as rosemary and chasteberry [3]. Extensive research has highlighted its multifaceted biological activities, encompassing anti-inflammatory [4], antioxidant [5], lipid-modulating [6], and hypoglycemic properties [7]. Our previous studies have underscored the beneficial effects of dietary UA supplementation in broilers [8] and finishing pigs [9]. UA influences the intestinal microbiome composition in mice and hamsters by modulating its formation while inhibiting pathogen proliferation and protecting against inflammation-induced oxidative stress damage [10,11]. Incorporating plant-based feed additives containing UA into diets represents an effective strategy to mitigate intestinal inflammation and enhance intestinal function [12]. In aquatic animals, the beneficial effects of dietary UA have only been confirmed in gilthead seabream (*Sparus aurata*): dietary 0.1% medicinal plant leaf extract (containing 10% UA) supplementation can increase weight gain and feed efficiency in gilthead seabream [13]. In largemouth bass (Micropterus salmoides) and rainbow trout, an intraperitoneal injection of UA can effectively inhibit virus infection and improve survival [14,15]. These results indicate that UA has potential application value in aquatic animals.

The largemouth bass, a prominent aquaculture species in China with significant economic value, contributed to a production output of 702,093 tons in 2021 [16]. Largemouth bass has been reported to exhibit susceptibility to intestinal tract diseases during the feeding process, which not only impairs intestinal functions but also reduces the digestibility and absorption of nutrients [17]. However, the effect of dietary UA on largemouth bass has not been reported. Therefore, this study aimed to investigate the effects of UA on the growth performance, antioxidant capacity, and intestinal health of largemouth bass and to determine the optimal supplemental level of UA in the largemouth bass diet so as to provide a theoretical basis and reference for the healthy breeding of this fish.

## 2. Materials and Methods

### 2.1. Experimental Design and Diets

Table 1 outlines the formulation and proximate composition of the experimental diets, featuring four distinct dietary groups supplemented with varying levels of UA. These groups consist of a control diet with 0 mg/kg UA (CON), and three diets with increasing UA concentrations of 250 mg/kg (UA250), 500 mg/kg (UA500), and 1000 mg/kg (UA1000). Prior to mixing, all ingredients were precisely weighed in their corresponding proportions and passed through a 60-mesh sieve for uniformity. The blended mixture was then pelletized into 1 mm diameter granules, subjected to air-drying, and stored in sealed plastic bags at 4 °C to preserve their quality until usage.

### 2.2. Experimental Fish and Feeding Management

All experimental protocols were approved by the Institutional Animal Care and Ethics Committee of Hunan Agricultural University, Changsha, China (protocol number 2024-00125), and the welfare of the fish was carefully considered. For this research, largemouth bass specimens were obtained from an aquaculture facility situated in Qichun, Hubei province, China. Before the formal experiment began, the test fish were first domesticated in the breeding system for 2 w, during which they were fed the basic diet (CON) until they were obviously full. After a two-week period of acclimatization, 800 healthy fish with an initial mean weight of approximately 11.0 g (with a standard deviation of 0.12 g) were randomly distributed among 20 net cages, each measuring 1.5 m by 1.5 m by 1.0 m. Each dietary group consisted of five replicate cages, each housing 40 fish. During the 8-week feeding trial, the daily feeding rate was 3% to 5% of the body weight, and this was adjusted according to the weather and feeding conditions. The fish were provided with feed twice daily, at 6:30 am and 4:30 pm. Stringent water quality measures were in place, ensuring that dissolved oxygen concentrations surpassed 6.0 mg/L, ammonia nitrogen levels remained below 0.1 mg/L, nitrite concentrations did not exceed 0.03 mg/L, and the pH was maintained within a range of 7.5 ± 0.5.

### 2.3. Sample Collection

Upon conclusion of the 8-week feeding trial, all fish underwent a 24 h fast to standardize conditions prior to data collection, making sure the fish were in a uniform state before sampling. Growth parameters were then determined by weighing and counting the fish in each net cage, and three fish per cage were randomly selected for an analysis of their whole-body proximate composition. These fish were anesthetized with tricaine methanesulfonate (MS-222, Solarbio, Beijing, China) at a concentration of 100 mg/L to ensure a humane sampling process. Furthermore, four fish from each net cage were selected for a morphological index assessment. The intestines were promptly dissected, segmented, and rapidly frozen in liquid nitrogen to preserve their integrity, followed by storage at −80 °C for subsequent biochemical analysis and gene expression studies. Specifically, one middle intestine sample per cage was preserved in 4% paraformaldehyde solution for a histological examination. Furthermore, two fish from each cage were randomly selected after 6 h of feeding, and the distal intestinal digesta were collected for a microbial community analysis.

### 2.4. Proximate Composition Analysis

The compositional analysis of both the experimental diets and the whole body of the fish, including moisture, crude protein, crude lipid, and ash content, adhered to the standardized protocols outlined by the Association of Official Analytical Chemists (AOAC) [18]. Moisture content was accurately measured by drying the samples in an oven at 105 °C until a constant weight was achieved. The Kjeldahl method, incorporating acid digestion and analysis using a semi-automatic Kjeldahl System, was employed to calculate crude protein levels, with the final value expressed as N × 6.25. Crude lipid content was quantified using the Soxhlet extraction technique. Lastly, crude ash content was determined through the incineration of the samples at 550 °C within a muffle furnace.

### 2.5. Biochemical Analysis

The middle intestine tissues underwent homogenization in a 1:9 tissue-to-saline ratio, followed by centrifugation at 4 °C and 3500 r /min for 10 min, to isolate the supernatants for subsequent analysis. Commercial kits (Nanjing Jiancheng Bioengineering Institute, Nanjing, China) were used to determine antioxidant-related indices, including total antioxidant capacity (T-AOC) (A015-2-1), T-SOD (A001-3) and MDA (A003-1), following the corresponding manufacturer’s instructions [19].

### 2.6. Intestinal Histological Analysis

The tissue samples, once fixed, underwent meticulous rinsing with PBS and water, repeating the process 3–4 times to eliminate any residual fixative. The samples were then carefully trimmed and dehydrated through a graded ethanol series. For optimal clearing, the samples were infiltrated with xylene and embedded in paraffin blocks. Employing a rotary microtome from Leica (Bayreuth, Germany), the paraffin blocks were precisely sectioned into five-micrometer-thick serial slices. These slices were mounted onto slides and subsequently stained with hematoxylin and eosin (H&E), adhering to the methodology outlined by Mi et al. [20].

### 2.7. Quantitative Real-Time PCR Analysis

The quantification of intestinal barrier-related gene mRNAs, including claudin-1, occludin, zonula occluden-*1 (zo-1*), interlenkin 8 (*il-8*), interlenkin 1β (*il-1β*), interlenkin 10 (*il-10*), and tumor necrosis factor-α (*tnf-α*), was achieved via RT-qPCR. This involved an initial extraction of total RNA from the tissue samples using a SteadyPure RNA extraction kit (Ecoray Biology, Changsha, Hunan, China). The extracted RNA was then converted to cDNA with the aid of an Evo M-MLVA reverse transcription kit (Ecoray Biology, Hunan). Subsequently, the abundance of these specific mRNAs was determined using a quantitative PCR kit (AG11701, Ecoray Biology, Hunan). Custom-designed primers specific to the target genes were synthesized by Beijing Qingke Biotechnology Co., Ltd., Changsha, China, and their sequences are listed in Table 2. To standardize the gene expression data, *ef1α* served as the internal reference gene. Finally, the 2^−ΔΔCt^ method was applied to analyze the gene expression levels, enabling a comparative evaluation of the relative expression patterns of the target genes among various samples [21].

### 2.8. Intestinal Microbiota Analysis

An E.Z.N.A. Microbial DNA Kit (Omega Bio-tek, Inc., Norcross, GA, USA) was used to extract microbial genomic DNA according to the instructions. The V3-V4 regions of the bacteria 16S rDNA genes were amplified via PCR using 338F/806R barcoded primers (Applied Biosystems, Carlsbad, CA, USA). The PCR products were analyzed using an NEB Next Ultra II DNA Library Prep Kit (New England Biolabs, Inc., Ipswich, MA, USA). Finally, the library was sequenced on the Illumina Miseq/Novaseq 6000 (Illumina, San Diego, CA, USA) platform, and the raw data obtained from sequencing were submitted to the NCBI SRA database. After the data were split and spliced, short sequences were filtered and removed. Qualified sequences were clustered into operational taxonomic units (OTUs) at a similarity threshold of 97% using the Uparse algorithm of Vsearch (v2.7.1) software. QIIME (v1.8.0) was used to generate rarefaction curves and to calculate the richness and diversity indices based on the OTU information, and R (v3.6.0) software was used for plotting. A partial least squares discriminant analysis (PLS-DA) was conducted to graphically visualize the differences in the bacterial composition among the groups, using the R language package “mixOmics” [22].

### 2.9. Statistical Analysis

All statistical analyses were conducted using SPSS 23.0 (IBM Corp., Chicago, IL, USA). Data were tested via homogeneity of variance (Levene’s test) and then analyzed using a one-way analysis of variance (ANOVA) with Duncan’s multiple-range tests. The results are presented as means ± standard error (SEM), and *p* < 0.05 was considered to indicate significant differences. The data were visualized using GraphPad Prism 8. Furthermore, a follow-up trend analysis was conducted using orthogonal polynomial contrasts to determine the significant effects (linear and/or quadratic).

## 3. Results

### 3.1. Growth Performance

**Figure 1:**
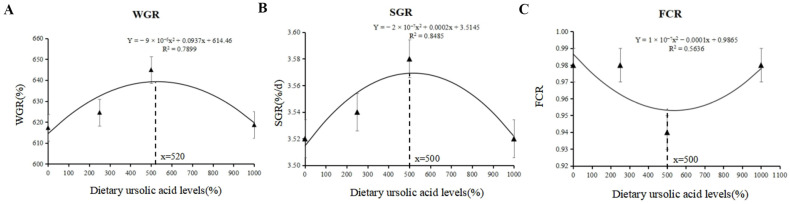
The optimum inclusion levels of UA based on specific growth rate (**A**), weight gain rate (**B**), and feed conversion ratio (**C**) in largemouth bass.

As shown in Table 3, the final body weight (FBW), weight gain rate (WGR), and specific growth rate (SGR) in the UA500 group were significantly higher than those in the other groups (*p* < 0.05). The feed conversion ratio (FCR) in the UA500 group significantly decreased (*p* < 0.05), and the hepatosomatic index (HSI) in the UA supplemental groups significantly decreased (*p* < 0.05). Additionally, there were no significant differences in the survival rate (SR), Fulton’s condition factor (CF), visceral somatic index (VSI), protein retention rate (PRR), or lipid retention rate (LRR) among all groups (*p* > 0.05). According to the regression analysis, the optimum UA inclusion level was 520 mg/kg based on WGR (Figure 1A) and 500 mg/kg based on SGR (Figure 1B) and FCR (Figure 1C).

### 3.2. Intestinal Antioxidant Capacity

**Figure 2:**
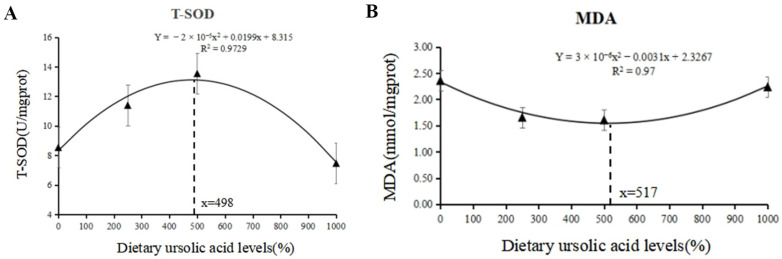
The optimum inclusion levels of UA based on T-SOD (**A**) and MDA (**B**) in largemouth bass.

As shown in Table 4, no statistical difference (*p* > 0.05) was observed in T-AOC among all groups. UA supplementation significantly increased T-SOD activity, which reached the highest level in the UA500 group and had quadratic effects (*p* < 0.001). According to the regression analysis, the optimum UA inclusion level was 498 mg/kg based on T-SOD (Figure 2A) and 517 mg/kg based on MDA (Figure 2B).

### 3.3. Intestinal Physical Barrier

**Figure 3:**
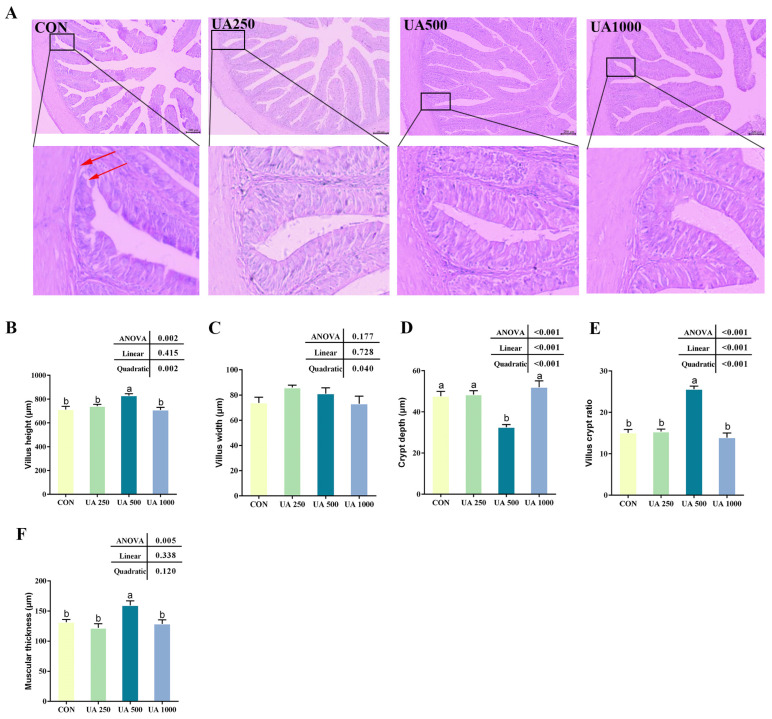
Effects of UA supplementation on intestinal morphology. (**A**) Hematoxylin and eosin (H&E) staining for histology examination (scale bar = 200 μm). (**B**) Villus height. (**C**) Villus width. (**D**) Crypt depth. (**E**) Villus crypt ratio. (**F**) Muscular thickness. Results are presented as the means (±SEM) (*n* = 5). Bars with different letters are significantly different (Duncan’s test; *p* < 0.05). The red arrows indicate structural deterioration, lax basement membrane, and infiltration of inflammatory cells. CON, the control diet; UA250, the CON diet supplemented with 250 mg/kg UA; UA500, the CON diet supplemented with 500 mg/kg UA; UA1000, the CON diet supplemented with 1000 mg/kg UA.

The intestines of the fish in the CON group exhibited a loosening of the basement membrane and an infiltration of inflammatory cells. Conversely, the UA500 group demonstrated an enhancement in intestinal structure and a reduction in inflammatory cell infiltration when compared with the CON group (Figure 3A). Furthermore, the UA500 group displayed a noteworthy augmentation in villus height, the villus–crypt ratio, and muscular thickness in contrast to the control group, as well as a significant decrease in crypt depth (*p* < 0.05, Figure 3B–F).

**Figure 4:**
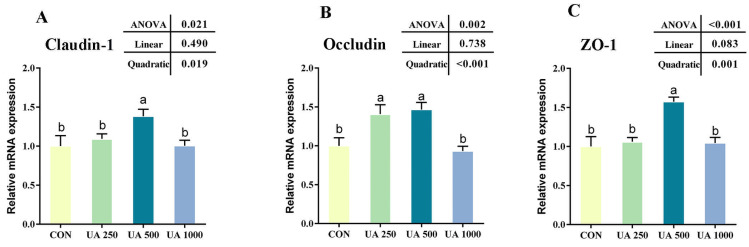
Effects of UA supplementation on relative expression of genes related to intestinal tight junctions. (**A**) *claudin-1*. (**B**) *occludin*. (**C**) Zona occluden-1 (*zo-1*). Results are given as the means (±SEM) (*n* = 5). Bars with different letters are significantly different (Duncan’s test; *p* < 0.05). CON, the control diet; UA250, the CON diet supplemented with 250 mg/g UA; UA500, the CON diet supplemented with 500 mg/kg UA; UA1000, the CON diet supplemented with 1000 mg/kg UA.

As shown in Figure 4, the results indicate that, in the UA250 group, there was no marked influence on the mRNA relative expressions of *zo-1* and *claudin-1* (*p* > 0.05, Figure 4A,C), but the mRNA relative expression of occludin increased compared with that in the CON group (*p* < 0.05, Figure 4B). Moreover, the mRNA relative expressions of *claudin-1*, *occludin*, and *zo-1* in the UA500 group clearly increased compared with those in the control group (*p* < 0.05, Figure 4); those in the UA1000 group did not change compared with those in the CON group.

### 3.4. Intestinal Immunological Barrier

**Figure 5:**
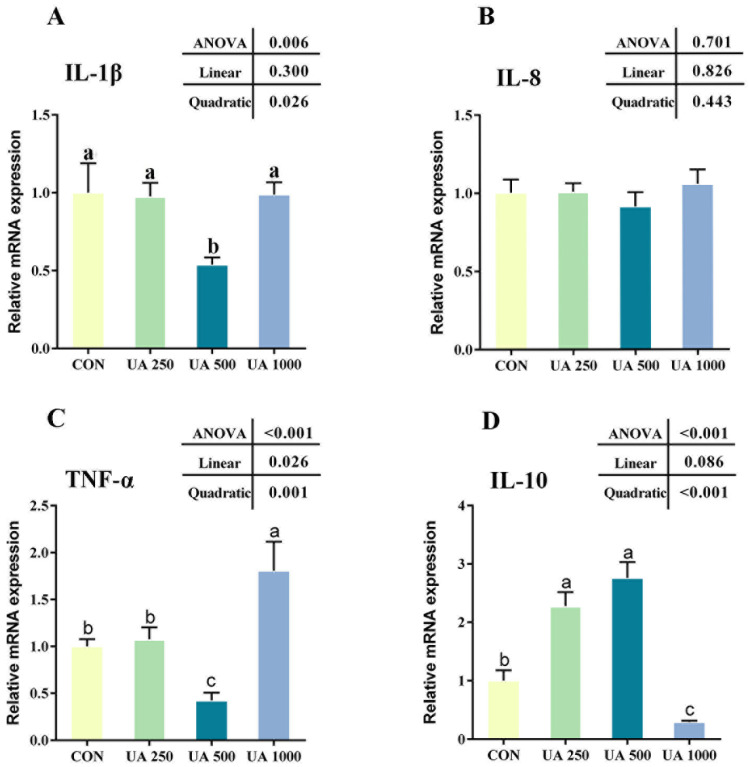
Intestinal expression levels of inflammation-related genes in largemouth bass fed with different levels of UA. (**A**) Interleukin-1β (*il-1β*). (**B**) Interleukin-8 (*il-8*). (**C**) Tumor necrosis factor-α (*tnf-α*). (**D**) Interleukin-10 (*il-10*). Results are given as the means (±SEM) (*n* = 5). Bars with different letters are significantly different (Duncan’s test; *p* < 0.05). CON, the control diet; UA250, the CON diet supplemented with 250 mg/kg UA; UA500, the CON diet supplemented with 500 mg/kg UA; UA1000, the CON diet supplemented with 1000 mg/kg UA.

As shown in Figure 5, the mRNA relative expressions of the pro-inflammatory genes *il-1β* and *tnf-α* significantly decreased in the UA500 group compared with the CON group (*p* < 0.05, Figure 5A,C), while the mRNA relative expression of *tnf-α* significantly increased in the UA1000 group (*p* < 0.05, Figure 5C); those in the UA250 group did not change compared with those in the CON group. Compared with the CON group, the mRNA relative expression of the anti-inflammatory gene *il-10* significantly increased in the UA250 and UA500 groups (*p* < 0.05, Figure 5D) and significantly decreased in the UA1000 group (*p* < 0.05, Figure 5D).

### 3.5. Intestinal Microbiota (Microbiological Barrier)

Approximately 2,182,513 sequences were obtained from the largemouth bass intestinal microbiota using high-throughput sequencing. An alpha diversity analysis of the intestinal microbiota revealed that dietary UA had a significant effect on OTU diversity (Table 5). The results show that there were no significant differences in the Chao1, observed_species, PD_whole_tree, Shannon, or Simpson indices among all groups (Table 5, *p* > 0.05).

**Figure 6:**
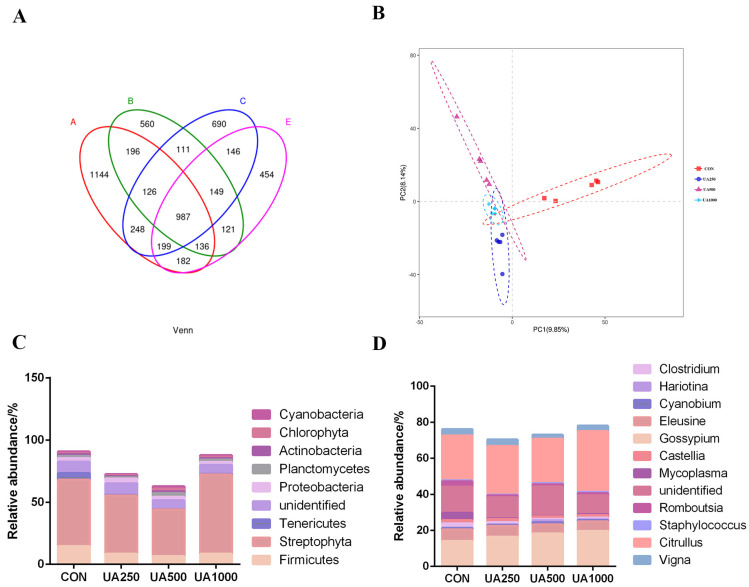
Intestinal microbiota in largemouth bass fed with different levels of UA. (**A**) Venn diagram. (**B**) Partial least squares discrimination analysis (PLS-DA). (**C**) Microbiota composition at the phylum level with relative abundance in the top eight. (**D**) Microbiota composition at the genus level with relative abundance in the top twelve. Letters represented the different trial groups in Figure 6A: A, CON; B, UA250; C, UA500; E, UA1000.

A Venn analysis revealed that 987 OTUs were shared among all groups, and the UA500 group had the most unique microbes (2656 OTUs) (Figure 6A). Furthermore, a partial least squares discrimination analysis (PLS-DA) and an anoism analysis of the microbial flora found that the intestinal microbial community in the UA1000 group was significantly different (*p* = 0.022) from that in the CON group (Figure 6B; Table 6).

At the phylum level (Figure 6C; Table 7), Streptophyta, Firmicutes, and Tenericutes were the dominant phyla in the intestinal microbiota of the largemouth bass. Compared with the CON group, the UA groups exhibited a significant decrease in the abundance of Firmicutes (*p* < 0.05). In addition, compared with the CON group, the relative abundance of Tenericutes in the UA500 group significantly increased, while Proteobacteria significantly decreased (*p* < 0.05).

At the genus level (Figure 6D; Table 8), the dominant genera were *Citrullus*, *Gossypium*, and *Unidentified*. Compared with the CON group, the relative abundance of *Mycoplasma* significantly decreased in the UA supplementation groups (*p* < 0.05). Additionally, the relative abundance of *Cyanobium* significantly decreased in the UA250 and UA500 groups (*p* < 0.05). Interestingly, in the UA250 group, the relative abundance of *Staphylococcus* significantly decreased (*p* < 0.05), while in the UA500 group, the relative abundance of *Clostridium* significantly increased (*p* < 0.05).

### 3.6. Correlation Analysis between Intestinal Microbiome and Intestinal Barrier Gene Expression

**Figure 7:**
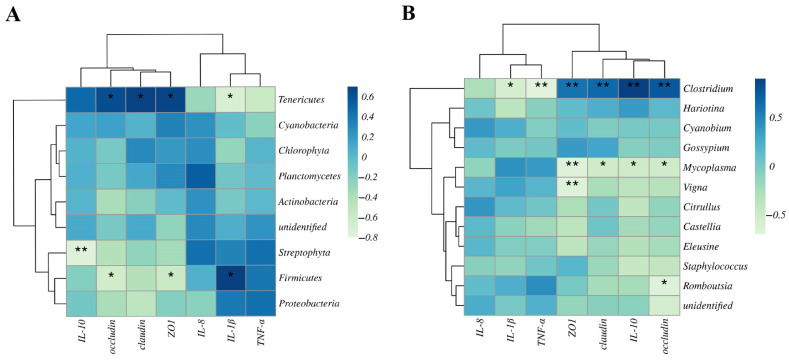
Correlation analysis between intestinal microbiome and intestinal barrier gene expression. (**A**) Heatmap of Spearman’s correlation between intestinal barrier gene expression and intestinal microbiota at the phylum level. (**B**) Heatmap of Spearman’s correlation between intestinal barrier gene expression and intestinal microbiota at the genus level. Significant correlations are marked by * *p* < 0.05, ** *p* < 0.01.

As illustrated in Figure 7A, microbial phyla, including Tenericutes, Streptophyta, and Firmicutes, showed a significant correlation with the mRNA expression of intestinal barrier genes. Tenericutes had a positive linear correlation with *claudin-1*, *occludin*, and *zo-1* (*p* < 0.05) while showing a negative linear correlation with *il-1β* (*p* < 0.05). Firmicutes exhibited a significant negative linear correlation with the expression levels of *occludin* and *zo-1* (*p* < 0.05) while showing a positive linear correlation with *il-1β* (*p* < 0.05). Moreover, *Mycoplasma*, *Vigna*, and *Romboutsia* showed a significant correlation with the mRNA expression of intestinal barrier genes (Figure 7B). *Mycoplasma* exhibited a significant negative linear correlation with the expression levels of *claudin-1*, *occludin*, *il-10* (*p* < 0.05), and *zo-1* (*p* < 0.01).

## 4. Discussion

The current literature lacks sufficient reports on the effect of UA on aquatic animals. Salomón et al. [13] showed that dietary 0.1% medicinal plant leaf extract (containing 10% UA) supplementation increased weight gain and feed efficiency in gilthead seabream (*Sparus aurata*). Similarly, this study demonstrated that UA supplementation increased both largemouth bass growth performance and feed efficiency. The optimal UA supplementation levels for maximum largemouth bass growth and feed efficiency were determined to be 520 mg/kg and 500 mg/kg, respectively. However, our previous study found that UA supplementation had no significant effects on the growth performance or carcass quality in finishing pigs but improved intestinal health, as indicated by the prevalence of *Prevotella* [9]. Similarly, dietary UA did not significantly affect the growth performance of yellow-feathered broilers but improved feed efficiency and slaughter performance [23]. We speculated that these different results regarding growth performance may be primarily attributed to variations in UA addition levels and experimental animal models. It has been reported that a lower hepatosomatic index is beneficial for fish health [24]. In line with this notion, our results revealed that the largemouth bass fed a diet supplemented with UA exhibited a significant reduction in the hepatosomatic index compared with the CON group.

Multiple investigations have underscored the antioxidative potential of UA, demonstrating its ability to mitigate oxidative stress [25,26,27]. Despite the common assumption that UA and related triterpenoids lack the phenolic motifs necessary for direct oxygen species (ROS) scavenging and metal ion chelation, recent findings have challenged this notion, albeit acknowledging their somewhat limited direct scavenging effect [28]. The activation of the nuclearfactorerythroid-2-relatedfactor2 (*nrf2)/heme oxygenase (ho-1)* signaling pathway represents a crucial mechanism in combating oxidative stress. UA has the ability to enhance the expression of *nrf2* and *ho-1* in HaCaT cells, thereby augmenting SOD activity and exerting antioxidant effects [29]. Our research revealed that dietary supplementation with UA at doses of 250 mg/kg and 500 mg/kg enhances T-SOD activity and reduces MDA levels, suggesting UA’s potential to facilitate *nrf2* translocation from the cytoplasm to the nucleus. This process promotes *ho-1* gene transcription, thereby amplifying SOD activity and bolstering the body’s antioxidant defenses, in line with previous observations by Kobayashi et al. [30].

The intestine serves as the primary site for nutrient absorption and digestion. Previous studies have demonstrated that enhancing the height and/or width of microvilli can expand the intestinal absorption area, thereby enhancing nutrient uptake capacity [31]. In this particular investigation, dietary supplementation with 500 mg/kg UA resulted in a significant increase in villus height, indicating that the enhanced growth performance and feed efficiency observed in the UA500 group may be attributed to an augmented intestinal absorption area facilitating superior nutrient assimilation. The improvement in intestinal villus height may be attributed to the fact that UA is an activator of Takeda G protein-coupled receptor 5 (*tgr5*) in vivo [32], which can inhibit the expression of apoptosis gene B-cell lymphoma-2 (*bcl-2*) by enhancing the phosphorylation of serine/threonine kinase and promoting epithelial cell survival [33]. Tight junction proteins, such as the cytoplasmic protein *zo-1* and transmembrane proteins, including claudins and occludin, are also implicated in the regulation of intestinal barrier function [34,35]. Prior investigations have highlighted the positive influence of the upregulation of *occludin* and *zonula occludens* gene expression on enhancing intestinal structure and barrier resilience in fish species [36]. UA supplementation increased the expression of *claudin-1* and *occludin* in the ileum of rats with CCl_4_-induced hepatic fibrosis [37,38]. Consistent with these findings, our study revealed an increase in *claudin-1*, *occludin*, and *zo-1* in the largemouth bass fed a diet supplemented with 500 mg/kg UA. These results suggest that dietary UA improves intestinal barrier function by enhancing its physical integrity and reducing permeability, thereby promoting intestinal health in fish.

The inflammatory cascade in aquatic organisms is intricately governed by cytokines such as *il-1β*, *tnf-α*, *il-8*, and *il-10*, which play pivotal roles in modulating inflammatory responses and immune functions [39]. Extensive research, both in vivo and in vitro, has consistently demonstrated that UA exerts a notable suppressive effect on the expression of pro-inflammatory cytokines, such as *il-1β*, *il-6*, *il-8*, and *tnf-α* [40,41]. In line with these findings, our study revealed that dietary supplementation with 500 mg/kg UA significantly decreased the intestinal expression of the pro-inflammatory genes *il-1β* and *tnf-α* while concurrently enhancing the expression of the anti-inflammatory gene *il-10* at both 250 mg/kg and 500 mg/kg doses. These results suggest that supplementation with an appropriate amount of UA can effectively mitigate intestinal inflammation. The dosage of UA appears to be a critical factor influencing its immunomodulatory effects. Previous studies, including a study by Zhao et al. [42], have highlighted that lower doses of UA (<25 mg/kg) tend to be more efficacious, while higher doses (>50 mg/kg) may not only fail to provide therapeutic benefits but could also potentially exacerbate inflammation in mice. Consistent with our study, a high dose (1000 mg/kg) of UA did not suppress the inflammatory response. Interestingly, a previous study suggested that a high dose of UA induced ROS production and activated ERK1/2 and p38 MAPK pathways, causing an increase in *il-1β* release [43]. In our study, a high dosage (1000 mg/kg) of UA exhibited no impact on *il-1β*. However, it upregulated the expression of *tnf-α*, downregulated the expression of *il-10*, and facilitated the intestinal inflammatory response. These discrepancies could potentially be attributed to factors such as the bioavailability of UA and animal species and their physiological stages, which still need further research.

The intestinal microbiota can produce harmful compounds that lead to barrier dysfunction and disease development while also producing beneficial metabolites (such as SCFAs) with anti-inflammatory, antioxidant, and intestinal barrier repair functions that impact host health or disease progression [44]. Dietary patterns significantly influence the composition and functional capabilities of this microbial community. Previous studies have highlighted a dose–response decrease in the Shannon diversity index among proximal intestinal microbiota in mice exposed to UA, stemming from its antimicrobial activities [45]. Furthermore, a β-diversity analysis underscored pronounced variations in the intestinal microflora structure between the UA1000 treatment group and the control group. An analysis of the intestinal flora composition revealed that Streptophyta, Firmicutes, and Proteobacteria were the predominant phyla in the intestinal microbiota of all groups, consistent with the findings of previous studies on largemouth bass [46,47]. In a correlation analysis at the phylum level, it was found that Firmicutes was negatively correlated with physical barrier gene expression and positively correlated with inflammatory factor gene expression. Conversely, Tenericutes exhibited the opposite pattern. Studies have demonstrated that the elevation of Firmicutes is associated with the promotion of obesity, resulting in compromised intestinal barrier function and the onset of inflammation, whereas Tenericutes may enhance intestinal barrier function through fermentation metabolism for organic acid production [48]. Our study demonstrated that UA decreased the relative abundance of Firmicutes, indicating its potential involvement in regulating the inflammatory response in largemouth bass. Recent studies have demonstrated that dietary supplementation with propionate enhances the barrier integrity of aquatic animal intestines while effectively reducing intestinal inflammation [49]. Therefore, it can be speculated that dietary supplementation with UA may improve intestinal health by promoting an increase in the abundance of organic acid-producing bacteria, such as Tenericutes. Proteobacteria are Gram-negative bacteria belonging to a major bacterial branch encompassing various pathogenic species, such as *E. coli*, *Salmonella*, and *Helicobacter pylori*. In certain intestinal environments, increased Proteobacteria content serves as a microbial marker for intestinal diseases and inflammation [50,51]. Our study found that the abundance of Proteobacteria in the UA500 group decreased significantly, while that of Tenericutes increased. Hypoxia stress induced similar alterations in the composition of intestinal proteobacteria and Tenericutes in Cobia [52] and the Chinese black sleeper (*Bostrichthys sinensis*) [53]. At the genus level, *Cyanobium* is widely distributed in various aquatic environments; it enters the fish body through water ingestion during feeding and produces toxins that can induce liver damage, intestinal injury, and nervous system impairment in fish [54]. As the smallest and simplest prokaryote, *Mycoplasma* can elicit cytotoxicity and provoke a robust inflammatory response via hydrogen peroxide release and exotoxin production [55]. Moreover, most *Staphylococci* are known to be pathogenic, particularly *Staphylococcus* aureus, which has been associated with autoimmune diseases and excessive inflammation [56]. Our study found that diets supplemented with UA significantly decreased the abundance of *Cyanobium*, *Mycoplasma*, *Staphylococcus,* and *Clostridium*. However, *Mycoplasma*, belonging to the Firmicutes family, was negatively correlated with *claudin-1*, *occludin*, *il-10*, and *zo-1*. *Mycoplasma*, which has been reported to be highly associated with intestinal barrier function, was found to be increased in the intestinal mucosa of trout fed a high-starch diet [57]. These results suggest that the reduction in Tenericutes by UA may potentially benefit the intestinal health of largemouth bass, while the Firmicutes family (such as *Mycoplasma*) may be a target to enhance the intestinal barrier function. UA might improve both the physical and immune barriers of the intestine by lowering these harmful intestinal microbes.

## 5. Conclusions

In conclusion, dietary supplementation of UA significantly enhanced the growth performance and antioxidant capacity in largemouth bass while improving intestinal barrier function through its influence on the abundance of intestinal flora such as Tenericutes, Firmicutes, and *Mycoplasma*. Moreover, optimal dietary UA levels for largemouth bass were determined to be between 498 and 520 mg/kg based on quadratic regression analyses of WGR, SGR, and FCR or T-AOC and MDA content. However, a high level of UA in diets, such as 1000 mg/kg, significantly disrupted the intestinal physical, immune, and microbial barriers of largemouth bass.

## Figures and Tables

**Table 1:** Formulation and compositions of experimental diets (air-dry basis, %).

Item	Groups			
	CON	UA250	UA500	UA1000
Ingredients ^1^				
Peru fish meal	40.00	40.00	40.00	40.00
Chicken powder	10.00	10.00	10.00	10.00
Soybean meal	9.03	9.03	9.03	9.03
Gluten powder	7.00	7.00	7.00	7.00
Dephenolic cottonseed protein	5.00	5.00	5.00	5.00
Flour	6.00	6.00	6.00	6.00
Tapioca flour	5.00	5.00	5.00	5.00
Microcrystalline cellulose	6.55	6.30	6.05	5.55
Ursolic acid ^2^	0.00	0.25	0.50	1.00
Fish oil	6.50	6.50	6.50	6.50
Shrimp paste	2.00	2.00	2.00	2.00
Ca(H_2_PO_4_)_2_	1.00	1.00	1.00	1.00
Lysine	0.50	0.50	0.50	0.50
Methionine	0.10	0.10	0.10	0.10
Choline chloride	0.40	0.40	0.40	0.40
Vitamin premix ^3^	0.25	0.25	0.25	0.25
Mineral premix ^4^	0.50	0.50	0.50	0.50
Vitamin C	0.10	0.10	0.10	0.10
DMPT (C_5_H_11_SO_2_Br)	0.05	0.05	0.05	0.05
Ethoxyquinoline	0.02	0.02	0.02	0.02
Total	100	100	100	100
Nutrient composition ^5^				
Crude protein	49.10	50.05	50.25	49.56
Crude lipid	10.86	11.13	10.52	10.92
Ash	11.83	11.65	11.76	11.96
Moisture	8.98	8.52	8.60	8.37

CON, the control diet; UA250, the CON diet supplemented with 250 mg/kg UA; UA500, the CON diet supplemented with 500 mg/kg UA; UA1000, the CON diet supplemented with 1000 mg/kg UA. ^1^ Provided by Wuhan Zhengda Aquatic Products Co., Ltd., Wuhan, China. ^2^ Provided by Hunan Jinhan Pharmaceutical Co., Ltd., Changsha, China. ^3^ Premix supplied the following vitamins (kg^−1^): inositol, 600 mg; vitamin A, 40 mg; vitamin D_3_, 0.06 mg; vitamin E, 200 mg; vitamin K_3_, 10 mg; vitamin B_1_ (thiamine), 15 mg; vitamin B_2_ (riboflavin), 25 mg; vitamin B_6_, 20 mg; pantothenic acid, 50 mg; vitamin B_3_ (nicotinic acid), 200 mg; biotin, 3.2 mg; vitamin B_12_, 0.1 mg; folic acid, 10 mg; vitamin C, 210 mg. ^4^ Premix supplied the following minerals (kg^−1^): Cu (CuSO_4_), 25 mg; Fe (FeSO_4_), 407 mg; Zn (ZnSO_4_), 50 mg; Mn (MnSO_4_), 36 mg; Se (Na_2_SeO_3_), 1.8 mg; Mg (MgSO_4_), 4 g. ^5^ Crude protein, crude lipid, ash, and moisture levels were measured values.

**Table 2:** Real-time PCR primer sequences.

Genes	Primer ^1^	Sequence	Product Size (bp)	GenBank Accession Number
*il-1β*	F:5′-3′	CGTGACTGACAGCAAAAAGAGG	166	XM 038733429.1
	R:5′-3′	GATGCCCAGAGCCACAGTTC		
*il-8*	F:5′-3′	CGTTGAACAGACTGGGAGAGATG	112	XM 038704088.1
	R:5′-3′	AGTGGGATGGCTTCATTATCTTGT		
*tnfα*	F:5′-3′	CTTCGTCTACAGCCAGGCATCG	161	XM 038710731.1
	R:5′-3′	TTTGGCACACCGACCTCACC		
*il-* *10*	F:5′-3′	CGGCACAGAAATCCCAGAGC	119	XM 038696252.1
	R:5′-3′	CAGCAGGCTCACAAAATAAACATCT		
*occludin*	F:5′-3′	GATATGGTGGCAGCTACGGT	198	XM_038715419.1
	R:5′-3′	TCCTACTGCGGACAGTGTTG		
*claudin-1*	F:5′-3′	CCAGGGAAGGGGAGCAATG	160	XM_038713307.1
	R:5′-3′	GCTCTTTGAACCAGTGCGAC		
*zo-1*	F:5′-3′	ATCTCAGCAGGGATTCGACG	208	XM 038701018.1
	R:5′-3′	CTTTTGCGGTGGCGTTGG		
*ef1α*	F:5′-3′	TGCTGCTGGTGTTGGTGAGTT	147	XM_054354427.1
	R:5′-3′	TTCTGGCTGTAAGGGGGCTC		

^1^ F, forward primer; R, reverse primer; *zo-1*, zonula occluden-1; *il-1β*, interlenkin 1β; *il-8*, interlenkin 8; *tnf-α*, tumor necrosis factor-α; *il-10*, interlenkin 10.

**Table 3:** Effects of UA supplementation on growth performance.

Items	UA Supplementation Levels/(mg/kg)				ANOVA	Linear	Quadratic
	CON	UA250	UA500	UA1000			
IBW (g) ^1^	11.01 ± 0.01	11.00 ± 0.00	11.01 ± 0.01	11.02 ± 0.01	0.564	-	-
FBW (g) ^1,a^	78.95 ± 0.63 ^b^	79.67 ± 1.10 ^b^	82.02 ± 0.26 ^a^	79.17 ± 0.21 ^b^	0.020	0.319	0.015
WGR (%) ^1,b^	617.36 ± 5.62 ^b^	624.62 ± 9.76 ^b^	645.00 ± 2.32 ^a^	618.73 ± 2.41 ^b^	0.010	0.365	0.011
SGR (%/d) ^1,c^	3.52 ± 0.01 ^b^	3.54 ± 0.02 ^b^	3.58 ± 0.01 ^a^	3.52 ± 0.01 ^b^	0.020	0.353	0.012
SR (%) ^1,d^	97.50 ± 0.79	97.00 ± 0.50	97.50 ± 1.58	96.50 ± 3.50	0.770	-	-
FCR ^1,e^	0.98 ± 0.01 ^a^	0.98 ± 0.01 ^a^	0.94 ± 0.00 ^b^	0.98 ± 0.01 ^a^	0.030	0.511	0.035
CF (g/cm^3^) ^2,f^	2.19 ± 0.04	2.17 ± 0.03	2.14 ± 0.02	2.18 ± 0.02	0.380	-	-
VSI (%) ^2,g^	9.88 ± 0.20	9.17 ± 0.20	9.59 ± 0.22	9.38 ± 0.24	0.223	-	-
HIS (%) ^2,h^	2.41 ± 0.11 ^a^	1.98 ± 0.06 ^c^	2.12 ± 0.08 ^bc^	2.15 ± 0.07 ^bc^	0.005	0.150	0.037
PRR (%) ^1,i^	40.65 ± 1.74	38.37 ± 1.37	41.83 ± 1.16	38.70 ± 2.03	0.427	-	-
LRR (%) ^1,j^	53.40 ± 1.02	52.21 ± 2.13	57.04 ± 1.43	57.05 ± 1.11	0.071	-	-

IBW: initial body weight; FBW: final body weight; WGR: weight gain rate; SGR: specific growth rate; SR: survival rate; FCR: feed conversion ratio; CF: Fulton’s condition factor; VSI: visceral somatic index; HSI: hepatosomatic index; PRR: protein retention rate; LRR: lipid retention rate; CON, the control diet; UA250, the CON diet supplemented with 250 mg/kg UA; UA500, the CON diet supplemented with 500 mg/kg UA; UA1000, the CON diet supplemented with 1000 mg/kg UA. ^1^ Values are means ± SEM (*n* = 5). Values in the same row with different superscripts represent statistically significant differences (*p* < 0.05). ^2^ Values are means ± SEM (*n* = 20). Values in the same row with different superscripts represent statistically significant differences (*p* < 0.05). ^a^ Final body weight (FBW, g) = final body weight/final number of fish. ^b^ Weight gain rate (WGR, %) = 100 × (final body weight − initial body weight)/initial body weight. ^c^ Specific growth rate (SGR, % day^−1^) = 100 × (Ln final individual weight − Ln initial individual weight)/number of feeding days. ^d^ Survival rate (SR, %) = 100 × (final number of fish)/(initial number of fish). ^e^ Feed conversion ratio (FCR) = feed consumed/weight gain. ^f^ Fulton’s condition factor (CF, g/cm^3^) = 100 × body weight/body length^3^. ^g^ Viscerosomatic index (VSI, %) = 100 × (viscera weight, g)/(whole bodyweight, g). ^h^ Hepatosomatic index (HSI, %) = 100 × hepatosomatic wet weight/body wet weight. ^i^ Protein retention rate (PRV, %) = 100 × [Final weight (g) × Final fish protein (%) − Initial weight (g) × Initial fish protein (%)]/[Feed intake (g) × Feed protein (%)]. ^j^ Lipid retention rate (LRR, %) = 100 × [Final weight (g) × Final fish lipid (%) − Initial weight (g) × Initial fish lipid (%)]/[Feed intake (g) × Feed lipid (%)].

**Table 4:** Effects of UA supplementation on intestinal antioxidant capacity.

Items	Ursolic Acid Supplemental Levels/(mg/kg)				ANOVA	Linear	Quadratic
	CON	UA250	UA500	UA1000			
T-AOC(mmol/gprot)	0.40 ± 0.03	0.41 ± 0.04	0.43 ± 0.05	0.39 ± 0.07	0.958	-	-
T-SOD (U/mgprot)	8.54 ± 0.39 ^b^	11.40 ± 1.03 ^a^	13.55 ± 0.66 ^a^	7.47 ± 0.67 ^b^	<0.001	0.768	<0.001
MDA (nmol/mgprot)	2.36 ± 0.14 ^a^	1.66 ± 0.08 ^b^	1.61 ± 0.16 ^b^	2.24 ± 0.19 ^a^	0.003	0.518	<0.001

Values are means ± SEM (*n* = 5). Values in the same row with different superscripts represent statistically significant differences (*p* < 0.05). CON, the control diet; UA250, the CON diet supplemented with 250 mg/kg UA; UA500, the CON diet supplemented with 500 mg/kg UA; UA1000, the CON diet supplemented with 1000 mg/kg UA.

**Table 5:** Effects of UA supplementation on intestinal microbial diversity.

Items	Ursolic Acid Supplemental Levels/(mg/kg)				ANOVA
	CON	UA250	UA500	UA1000	
chao1	1271.55 ± 10.23	1124.00 ± 49.05	1251.41 ± 27.78	1217.69 ± 43.49	0.148
observed_species	1047.00 ± 17.00	895.58 ± 36.05	1017.20 ± 34.48	995.78 ± 39.94	0.084
PD_whole_tree	182.19 ± 8.97	161.56 ± 10.00	157.27 ± 15.77	157.55 ± 10.66	0.525
shannon	4.51 ± 0.54	4.33 ± 0.26	4.21 ± 0.41	4.20 ± 0.18	0.918
Simpson	0.84 ± 0.04	0.84 ± 0.02	0.80 ± 0.04	0.81 ± 0.02	0.716

Values are means ± SEM (*n* = 5). CON, the control diet; UA250, the CON diet supplemented with 250 mg/kg UA; UA500, the CON diet supplemented with 500 mg/kg UA; UA1000, the CON diet supplemented with 1000 mg/kg UA.

**Table 6:** Anoism analysis of microbial flora.

Method Name	R Statistic	*p*-Value	Number of Permutations	Group
ANOSIM	−0.0600	0.680	999	CON-UA250
	0.1200	0.062	999	CON-UA500
	0.3360	0.022	999	CON-UA1000
	−0.0040	0.447	999	UA250-UA500
	0.0960	0.181	999	UA250-UA1000
	0.0960	0.155	999	UA500-UA1000
	0.0670	0.128	999	all

CON, the control diet; UA250, the CON diet supplemented with 250 mg/kg UA; UA500, the CON diet supplemented with 500 mg/kg UA; UA1000, the CON diet supplemented with 1000 mg/kg UA.

**Table 7:** The predominant intestinal bacterial phyla in largemouth bass fed with different levels of UA (%).

Items	Ursolic Acid Supplemental Levels/(mg/kg)				ANOVA
	CON	UA250	UA500	UA1000	
Firmicutes	14.56 ± 0.46 ^a^	8.13 ± 1.20 ^b^	6.60 ± 1.64 ^b^	8.47 ± 0.24 ^b^	0.020
Streptophyta	53.51 ± 4.30	46.71 ± 9.16	37.45 ± 6.70	63.73 ± 2.00	0.072
Tenericutes	16.35 ± 1.21 ^b^	20.96 ± 4.46 ^b^	42.02 ± 7.71 ^a^	23.78 ± 3.79 ^b^	0.012
unidentified	9.10 ± 3.10	9.45 ± 4.10	7.16 ± 1.79	7.18 ± 1.43	0.890
Proteobacteria	3.06 ± 0.24 ^a^	3.66 ± 0.28 ^a^	1.93 ± 0.20 ^b^	3.34 ± 0.46 ^a^	0.042
Planctomycetes	1.68 ± 0.11	1.17 ± 0.20	2.97 ± 0.69	2.02 ± 0.39	0.071
Actinobacteria	1.51 ± 0.18	1.00 ± 0.09	1.46 ± 0.33	1.29 ± 0.15	0.336
Chlorophyta	0.86 ± 0.06	0.67 ± 0.06	1.33 ± 0.24	1.11 ± 0.18	0.084
Cyanobacteria	1.34 ± 0.20	0.92 ± 0.16	1.84 ± 0.37	1.23 ± 0.13	0.074

Values are means ± SEM (*n* = 5). Values in the same row with different superscripts represent statistically significant differences (*p* < 0.05). CON, the control diet; UA250, the CON diet supplemented with 250 mg/kg UA; UA500, the CON diet supplemented with 500 mg/kg UA; UA1000, the CON diet supplemented with 1000 mg/kg UA.

**Table 8:** The predominant intestinal bacterial genera in largemouth bass fed with different levels of UA (%).

Items	Ursolic Acid Supplemental Levels/(mg/kg)				ANOVA
	CON	UA250	UA500	UA1000	
*Gossypium*	14.09 ± 1.29	16.44 ± 1.16	18.31 ± 1.51	19.70 ± 1.40	0.095
*Eleusine*	6.41 ± 0.40	6.08 ± 0.68	5.01 ± 0.62	5.32 ± 0.22	0.315
*Cyanobium*	0.83 ± 0.06 ^a^	0.49 ± 0.10 ^b^	0.44 ± 0.05 ^b^	0.66 ± 0.08 ^ab^	0.026
*Hariotina*	0.29 ± 0.07	0.31 ± 0.04	0.43 ± 0.12	0.42 ± 0.06	0.361
*Clostridium*	1.33 ± 0.19 ^b^	2.36 ± 0.60 ^ab^	2.92 ± 0.49 ^a^	1.08 ± 0.09 ^b^	0.045
*Castellia*	1.76 ± 0.16	1.71 ± 0.23	1.33 ± 0.22	1.37 ± 0.10	0.254
*Mycoplasma*	4.08 ± 1.15 ^a^	0.38 ± 0.11 ^b^	0.23 ± 0.11 ^b^	0.56 ± 0.38 ^b^	0.022
*unidentified*	14.52 ± 2.95	11.74 ± 3.94	16.32 ± 5.46	10.46 ± 1.06	0.783
*Romboutsia*	2.84 ± 0.42	2.56 ± 0.49	1.96 ± 0.31	1.97 ± 0.13	0.397
*Staphylococcus*	0.60 ± 0.05 ^a^	0.38 ± 0.10 ^b^	0.67 ± 0.03 ^a^	0.76 ± 0.02 ^a^	0.034
*Citrullus*	24.99 ± 1.97	27.06 ± 5.47	24.63 ± 3.68	33.78 ± 1.24	0.374
*Vigna*	3.49 ± 0.39	3.56 ± 0.55	2.21 ± 0.26	2.94 ± 0.21	0.076

Values are means ± SEM (*n* = 5). Values in the same row with different superscripts represent statistically significant differences (*p* < 0.05). CON, the control diet; UA250, the CON diet supplemented with 250 mg/kg UA; UA500, the CON diet supplemented with 500 mg/kg UA; UA1000, the CON diet supplemented with 1000 mg/kg UA.

## Data Availability

The data presented in this study are available on request from the corresponding author. The availability of the data is restricted to investigators based at academic institutions.
